# The risk of early mortality of polytrauma patients associated to ISS, NISS, APACHE II values and prothrombin time

**DOI:** 10.1186/1752-2897-7-6

**Published:** 2013-05-24

**Authors:** Ladislav Mica, Kaspar Rufibach, Marius Keel, Otmar Trentz

**Affiliations:** 1 Division of Trauma Surgery, University Hospital of Zürich, Zurich, Switzerland; 2 Division of Biostatistics, University of Zürich, Zurich, Switzerland; 3 University Hospital of Othopedic Surgery, Inselspital, Bern, Switzerland; 4 Department of Trauma Surgery, University Hospital of Zürich, Zurich, Switzerland

**Keywords:** Polytrauma, ISS, NISS, APACHE II, Prothrombin time

## Abstract

**Background:**

The early hemodynamic normalization of polytrauma patients may lead to better survival outcomes. The aim of this study was to assess the diagnostic quality of trauma and physiological scores from widely used scoring systems in polytrauma patients.

**Methods:**

In total, 770 patients with ISS > 16 who were admitted to a trauma center within the first 24 hours after injury were included in this retrospective study. The patients were subdivided into three groups: those who died on the day of admission, those who died within the first three days, and those who survived for longer than three days. ISS, NISS, APACHE II score, and prothrombin time were recorded at admission.

**Results:**

The descriptive statistics for early death in polytrauma patients who died on the day of admission, 1–3 days after admission, and > 3 days after admission were: ISS of 41.0, 34.0, and 29.0, respectively; NISS of 50.0, 50.0, and 41.0, respectively; APACHE II score of 30.0, 25.0, and 15.0, respectively; and prothrombin time of 37.0%, 56.0%, and 84%, respectively. These data indicate that prothrombin time (AUC: 0.89) and APACHE II (AUC: 0.88) have the greatest prognostic utility for early death.

**Conclusion:**

The estimated densities of the scores may suggest a direction for resuscitative procedures in polytrauma patients.

**Trial registration:**

*“Retrospektive Analysen in der Chirurgischen Intensivmedizin”*StV01-2008.

## Introduction

Advances in medicotechnical procedures have led to a significant improvement in the treatment of polytrauma patients. The standard resuscitative protocols (Advanced Trauma Life Support, ATLS®) are uniform and are applied to every patient who has suffered polytrauma. Trauma scoring systems, such as the Injury Severity Score (ISS), the New Injury Severity Score (NISS), and the Acute Physiology and Chronic Health Evaluation (APACHE II) score are often used to estimate the severity of trauma and the patients’ physiological health but are not used to predict the outcomes of polytrauma patients [1-3]. These parameters were chosen as independent predictive factors of death as previously shown [4]. The aim of this study was to show the distributions of the scores for these systems according to the time of death of polytrauma patients and to assess the prognostic quality of the three scores and their utility in providing orientation points for further decisions.

ISS and NISS describe the severity of tissue destruction. Both are defined with the same scoring system and both describe the severity of trauma based on anatomic region and injury pattern [1,2]. The injury pattern includes the severity of the trauma, and whether it increases the patient’s susceptibility to coagulopathy and subsequent systemic inflammatory response syndrome (SIRS) and other trauma-related diseases [5-7]. The anatomical injury directly indicated by ISS and NISS involves bleeding and leads to problems such as hypothermia and the coagulopathy of trauma shock [5]. It has been shown that coagulopathy develops in 98% of all patients with an ISS above 25 points, a pH value < 7.1, a core temperature < 34°C (93.2°F), or a systolic blood pressure < 70 mmHg [8]. There is clear evidence that most polytrauma (ISS ≥ 17) patients are admitted to the trauma center with already established coagulopathy [5]. A clear association has been demonstrated between coagulopathy and the early death of the patient but not between early death and prothrombin time at admission [9]. Coagulopathy is also a risk factor for the development of trauma-associated diseases, such as SIRS, and subsequent complications (multiorgan dysfunction syndrome and multiorgan failure) [10]. The APACHE II score is used to assess the patient’s physiological state, and may be pathologically elevated, reflecting the patient’s physiological health [3]. The distribution of these scoring systems according to the risk of death has not been shown yet. The ability to anticipate trauma complications based on these scoring systems could improve the survival rate in polytrauma patients. A reduction in trauma-associated diseases and therefore the reduction of intensive-care-unit (ICU) days could also economically benefit financially stressed social health-care systems. Better medical outcomes in polytrauma patients could reduce the social costs and shorten the patients’ rehabilitation to the workforce. In this study, we focused on widely used trauma scoring systems to assess the severity of trauma and used the prothrombin time as a parameter of coagulopathy. The aim was to show the distribution of widely used trauma scoring systems according to the death of the polytrauma patient.

## Material and methods

### Patients

Seven hundred seventy polytrauma patients admitted to the emergency department (1996–2006) of the University Hospital were included in this study. The admission criteria were ISS > 16 points, age ≥ 16 years. The patient sample was subdivided into three groups: those who died at admission, those who survived for 1–3 days, and those who survived for longer than three days. The objective was to analyse the independent predictors of the early death in polytrauma patients [4].

### Surgical treatment procedure

The surgical treatment of all patients followed the ATLS® guidelines and a previously established trauma-management protocol [11-13]. Briefly, after airway, ventilation and cardiovascular management, life-saving surgery was performed with decompression of the body cavities, hemorrhage control, and the removal of contaminated tissue. The first surgical interventions were followed by stabilization of the major fractures and radical debridement of dead tissue. In selected cases bleeding was stoped by interventional radiology. In all admitted patients, enteral nutrition was established within 24 h of trauma to avoid spontaneous transmigration of the enteral microbial flora and peritoneal contamination.

### Data collection

All the patients’ data were collected retrospectively. ISS, NISS, and APACHE II scores were calculated from the data collected at admission to the emergency department [1-3]. All data were retrieved from the patients’ records with the approval of the local Institutional Review Board (IRB) according to the University IRB guidelines and according to the declaration of Helsinki (*“Retrospektive Analysen in der Chirurgischen Intensivmedizin”* Nr. StV 01–2008).

### Trauma scoring systems

The ISS and NISS were used to define the severity of the trauma [1,2] based on AIS 2005 [14]. The APACHE II score was used to evaluate the overall physiological impairment of the patient [3].

### Laboratory parameters

The prothrombin time was measured at admission with a standardized method, as described previously [15].

### Statistical analysis

The descriptive statistics included frequencies and percentages for categorical data and medians (ranges) for continuous data. The diagnostic quality of the continuous variables was assessed using receiver operating characteristic (ROC) curves and the areas under the curves (AUCs). Wald confidence intervals for AUCs were computed on the logit scale and retransformed. To calculate density estimates for the scores, the density estimates under a log-concavity assumption were used leading to more precise results than using the mean-value only [16]. To describe the score distribution modes, estimates computed from the densities described above were used. The mode is the value on the x-axis at which the estimated density reaches its maximum. All computations were performed in R [17]. The log-concave density estimates were computed using the R package logcondens [18]. The statistical analysis was performed by the Institute for Biostatistics of the University of Zürich.

## Results

### Patient sample

In total, 770 patients with a median age of 39 years (range 16–89), who met the criterion for polytrauma (ISS ≥ 17) and were admitted to a level I trauma center, were included in this study; 573 were men and 197 were women. In total, 512 patients (66.5%) survived the first 72 h: 385 men (48.7% of all patients, 65.2% of men) and 127 women (16.4% of all patients, 64.5% of women) (Table 1). 84 patients suffered a penetrating trauma and 686 patients sufferd a blunt trauma.

**Table 1:** Characteristics of the patient sample (ISS: Injury Severity Score; NISS: New Injury Severity Score; APACHE II: Acute Physiology and Chronic Health Evaluation)

	**Sex male/female**	**N (% of all)**	**Age [a]**	**Prothrombin time [%]**	**ISS**	**NISS**	**APACHE II**
**Death day 0**	50/24	74 (9.6%)	47.5 (18–89, 47.7, ± 19.7)	37 (10–100, 42.1, ± 22.4)	41.0 (18–75, 40.3, ± 13.6)	50.0 (20–75, 52.0, ± 15.2)	30.0 (9–43, 29.0, ± 7.9)
**Death day 1-3**	138/46	184 (23.9%)	42.5 (17–89, 45.5, ± 19.2)	56 (10–100, 58.9, ± 23.6)	34.0 (17–75, 37.8, ± 13.4)	50.0 (20–77, 50.2, ± 14.1)	25.0 (6–44, 25.5, ± 7.6)
**Survival day > 3**	385/127	512 (66.5%)	36.5 (16–88, 39.1, ± 16.6)	84 (14–121, 80.5, ± 19.3)	29.0 (17–75, 32.6, ± 11.9)	41.0 (29–75, 41.2, ± 13.4)	15.0 (0–40, 15.2, ± 7.9)
**All**	573/197	770	38.5 (16–89, 41.4, ± 17.9)	78 (10–121, 73.7, ± 23.5)	33.0 (17–75, 34.6, ± 12.8)	43.0 (17–77, 44.4, ± 14.5)	19.0 (0–44, 18.9, ± 9.5)

Data are given as medians (range, mean, ±SD).

**Table 2:** The modes of the density estimates for prothrombin time, ISS, NISS, and APACHE II

	**Prothrombin time [%]**	**ISS**	**NISS**	**APACHE II**
**Death day 0**	28	32.5	58.8	33.2
**Death day 1-3**	53	29.2	50.6	22.9
**Survival day > 3**	95.4	25.1	31.6	17.5

### Distributions of ISS, NISS, APACHE II score, and prothrombin time

**Figure 1:**
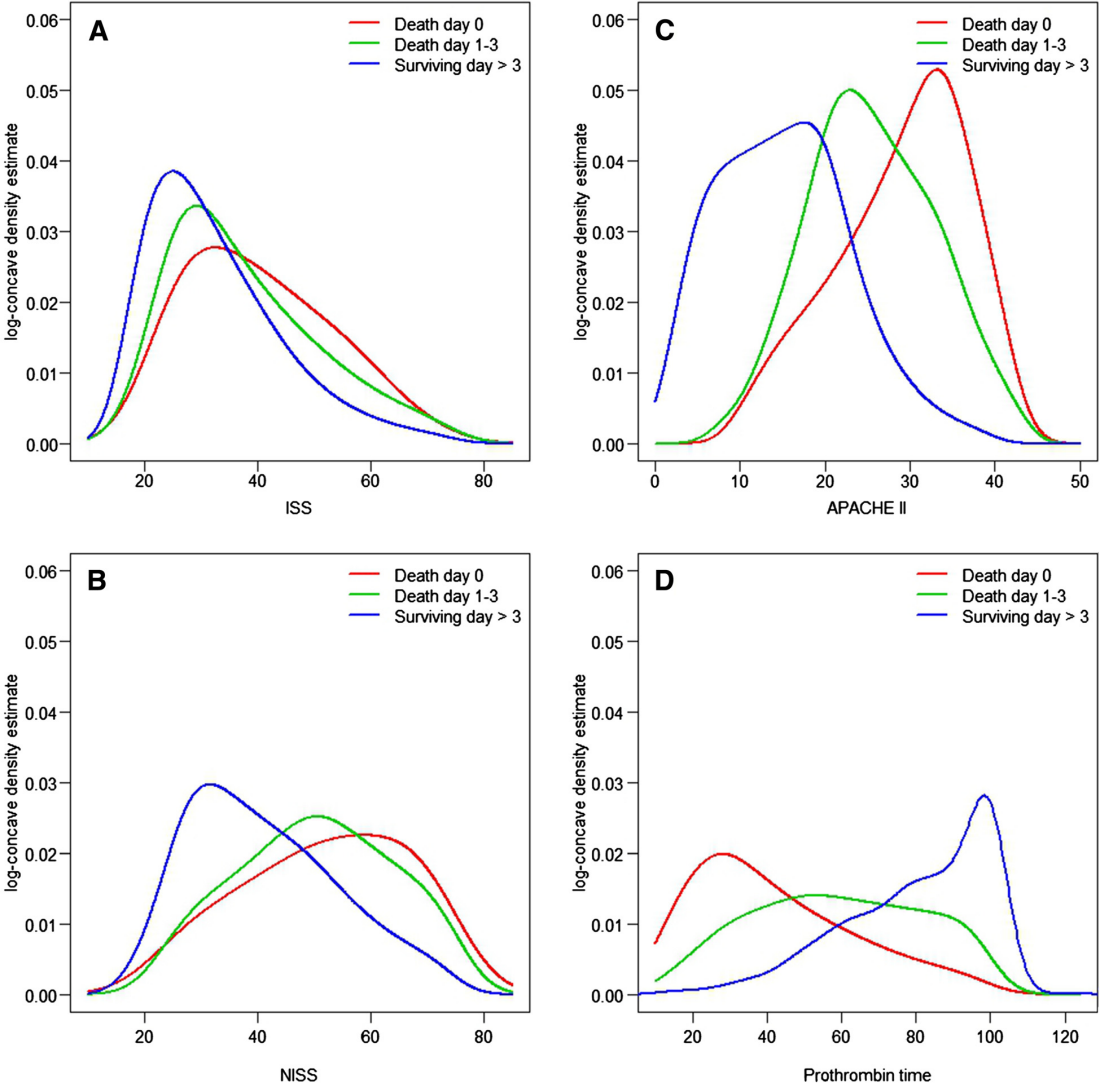
**Kernel density estimates for ISS (A), NISS (B), APACHE II (C), and prothrombin time (D). A:** The median ISS value for death at admission was 41.0 (27.0–50.0); followed by those for early death within the first 72 h (median 34.0 [25.0–45.0]) and for survival for > 3 days (median 29.0 [25.0–38.0]). **B:** The median NISS value for death at admission was 50.0 [41.0–66.0]), followed by those for patients who died within the first 72 h (median 50.0 [41.0–59.0]) and by those who survived for > three days (median 41.0 [29.0–50.0]). **C:** The median APACHE II score for death at admission was 30.0 (24.0–34.0), followed by those for patients who died within the first 72 h (median 25.0 [20.0–32.0]) and those who survived for > 3 days (median 15.0 [9.0–20.0]). **D:** The median prothrombin time for the patients who died on the day of admission was 37.0% (25.5–55.0%), increasing with increasing survival time (median 56% [42.0%–76.8%] for those surviving till days 1–3; median 84% [67.0%–100.0%] for those surviving for > 3 days).

From the density estimates, the mode of ISS was estimated for death on the day of admission (median 41.0, range 27.0–50.0, mode 32.5), for death within the first 72 h (median 34.0, range 25.0–45.0, mode 29.2), and for survival for > 3 days (median 29.0, range 25.0–38.0, mode 25.1) (Tables 1 and 2, Figure 1A). Similar distributions were estimated for NISS: patients who died at admission showed the highest values (median 50.0, range 41.0–66.0, mode 58.8), followed by the patients who died within the first 72 h (median 50.0, range 41.0–59.0, mode 50.6), and then by those who survived for > 3 days (median 41.0, range 29.0–50.0, mode 31.6) (Tables 1 and 2, Figure 1B). The density estimates for the APACHE II scores were highest in the patients who died at admission (median 30.0, range 24.0–34.0, mode 33.2), followed by those of the patients who died within the first 72 h (median 25.0, range 20.0–32.0, mode 22.9), and then by those of the patients who survived > 3 days (median 15.0, range 9.0–20.0, mode 17.5) (Tables 1 and 2, Figure 1C). Separate analysis of the prothrombin time at admission, as a part of the APACHE II scoring system, showed low values for those patients who died on the day of admission (median 37.0%, range 25.5%–55.0%) and those who died within the first 72 h (median 56%, range 42.0%–76.8%). Those patients who survived the first three days usually showed nonpathological prothrombin times (clinical norm > 75%, median 84%, range 67.0%–100.0%) (Tables 1 and 2, Figure 1D).

### Prognostic quality of ISS, NISS, APACHE II, and prothrombin time

**Figure 2:**
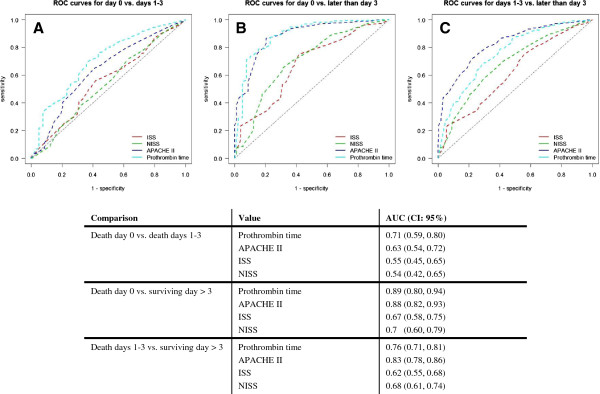
**Predictive quality of ISS, NISS, APACHE II and Prothrombin time. A:** ROC curves for prothrombin time, ISS, NISS, and APACHE II scores. Comparison of the values for patients who died on the day of admission vs patients who died within the first 72 h. **B:** ROC curves comparing the values for patients who died at admission and those who survived for more than three days. **C:** ROC curves comparing the values for patients who died within the first 72 h and those who survived for more than three days. AUC: area under the curve; CI, 95% confidence interval.

The values were transformed into ROC curves and the three groups compared. APACHE II and prothrombin time showed the highest predictive value in all three ROC analyses (Figure 2A–C). The ROC analysis indicated that the prothrombin time and APACHE II score most effectively predicted early death in polytrauma patients. For all parameters, the AUCs increased relative to those for the more distant points of death (Figure 2). These values were: prothrombin time (death day 0 vs surviving day > 3, AUC = 0.89 [CI, 0.80–0.94]; death day 0 vs death days 1–3, AUC = 0.71 [CI, 0.59–0.80]; and death days 1–3 vs surviving day > 3, AUC = 0.76 [CI, 0.71–0.81]); APACHE II score (death day 0 vs surviving day > 3, AUC = 0.88 [CI, 0.82–0.93]; death day 0 vs death days 1–3, AUC = 0.63 [CI, 0.54–0.72]; and death days 1–3 vs surviving day > 3, AUC = 0.83 [CI, 0.78–0.86]); ISS (death day 0 vs surviving day > 3, AUC = 0.67 [CI, 0.58–0.75]; death day 0 vs death days 1–3, AUC = 0.55 [CI, 0.45–0.65]; and death days 1–3 vs surviving day > 3, AUC = 0.62 [CI, 0.55–0.68]); and NISS (death day 0 vs surviving day > 3, AUC = 0.7 [CI, 0.60–0.79]; death day 0 vs death days 1–3, AUC = 0.54 [CI, 0.42–0.65]; and death days 1–3 vs surviving day > 3, AUC = 0.68 [CI, 0.61–0.74]) (Figure 2).

## Discussion

Tissue destruction, massive bleeding, and insufficient oxygenation after trauma are the main issues in emergency and trauma medicine. Polytrauma patients admitted to a level I trauma center usually already suffer coagulopathy, even when the transportation time is short. There is evidence that the disarrangement of the coagulation cascade occurs near the site of the accident [19-22]. Our aim was to assess the prognostic quality of several widely used trauma scoring systems and prothrombin time and their utility in providing some decision points for preclinical and early clinical trauma care. The definition of polytrauma was ISS > 16 points. However, no data are available on how the ISS, NISS and APACHE II are distributed in severely injured patients who suffer early death (within 72 h) [23].

### ISS and NISS: tissue destruction as a source of consumptive coagulopathy

ISS and NISS characterize the severity of injury according to the anatomic region, and indirectly indicate the mass of destroyed tissue in the patient [1,2]. Tissue destruction is associated with the activation of the kininogen–kallikrein system and releases Hageman factor (factor XII) [24]. The systemic activation of the blood coagulation cascade leads to the consumption of blood clotting factors. This consumption is reflected in ongoing bleeding and a reduced prothrombin time, which is the final common pathway of the intrinsic and extrinsic blood clotting pathways. This vicious cycle of consumptive coagulopathy and ongoing bleeding causes further bleeding and the classic ‘triad of death’ (coagulopathy, hypothermia, and acidosis), leading to the death of the patient. The data reported here indicate that the ISS is around 40 points and the NISS is around 50 in patients who die at admission. However, the ability to predict early death increases as more distant time points are compared (see Figure 2). This fact, demonstrated by the data, might reflect the early occurrence of complications other than exsanguination, e.g., transfusion-associated lung injury or early immunological reactions to trauma [23,25]. All patients with ISS > 16 and age ≥ 16 years were included in the study, with no consideration of the chronic state of their health. Some may have suffered more tissue destruction than others (see Figure 1A–B).

### APACHE II: physiological deterioration at admission

Parameters included in the APACHE II score are oxygenation, urinary excretion, and the acid–base system [3]. Tissue destruction in trauma patients leads to impaired microcirculation and hence to impaired oxygen supply. The resulting anaerobic glycolysis leads to acidosis and impairs the coagulation capacity of the blood. Cell destruction opens the intracellular K^+^ pool, and with the additional kidney damage resulting from trauma and free myoglobin, the potassium levels may rise. Anaerobic glycolysis reduces the cellular ATP concentration, resulting in the malfunction of the sodium–potassium ATPases, causing cellular edema, which maintains the vicious cycle of impaired microcirculation and ongoing cell damage. The loss of blood in trauma patients leads to secondary renal insufficiency and impaired excretion, which together with the accumulation of myoglobin in the renal tubules leads to reduced renal function [25]. In combination with shock, the ‘death triad’ of acidosis, coagulopathy, and hypothermia is maximally supported in polytrauma patients. The present data show three modes of the APACHE II scoring system based on survival time. Additional scoring according to age and chronic health may explain the three clearly separated modes of the APACHE II score-based survival time (Figure 1), even when all patients over the age of 16 years were included, with no distribution into age groups.

### Prothrombin time: reflecting the acute coagulopathy of trauma shock at admission

The acute coagulopathy of trauma shock is recognized as a very early event after trauma. Patients admitted to a trauma center already suffer coagulopathy, even when admission times are very short. Coagulopathy is likely to occur in 98% of patients with ISS > 25, pH < 7.1, a core temperature < 34°C, or systolic blood pressure < 70 mmHg [8]. This traditional description assumes that acute coagulopathy of trauma shock is a later event, mainly caused by resuscitative attempts. However, patients are admitted to the emergency department with already established or evolving coagulopathy resulting directly from trauma [26]. Similar cases were reported in a retrospective study of 1088 patients, which was confirmed by other retrospective studies based on large patient samples [21,22,26]. In these studies, a strong association was demonstrated between coagulopathy and mortality. Coagulopathy was also identified as an independent risk factor for acute renal failure and multiorgan failure, and was associated with a trend toward acute respiratory distress syndrome [26,27]. The prognostic quality of the prothrombin time increases as more distant points in time are compared (Figure 2). A possible interpretation is that the severity of trauma correlates with the severity of tissue destruction and the amounts of procoagulative factors released, as mentioned earlier [24]. However, the present data suggest that the prothrombin time at admission may be a good diagnostic marker of early death in polytrauma patients.

## Conclusions

The analysed scoring systems and protrombin time as independent predictors of the early death in polytrauma patients [4] revealed APACHE II and prothrombin time to have the highest prognostic quality in polytrauma patients for a hazzardous outcome. The shown modes of each single factor should provide an orientation help in resuscitative procedures.

## Competing interest

The authors declare that they have no competing interests.

## Authors’ contributions

All authors contributed equally to this work. LM carried out the idea and manuscript preparation. KR made the statistical analysis. MK and OT provided intellectual support and the acquisition of data. All authors read and approved the final manuscript.
